# Medial meniscus posterior root tears with advanced osteoarthritis or subchondral insufficiency fracture are good indications for unicompartmental knee arthroplasty at a minimum 2-year follow-up

**DOI:** 10.1007/s00402-024-05671-1

**Published:** 2024-12-18

**Authors:** Koki Kawada, Yusuke Yokoyama, Yuki Okazaki, Masanori Tamura, Toshifumi Ozaki, Takayuki Furumatsu

**Affiliations:** 1 Department of Orthopaedic Surgery, Okayama University Graduate School of Medicine, Dentistry, and Pharmaceutical Sciences, Okayama, Japan; 2 Department of Orthopaedic Surgery, Okayama Red Cross Hospital, 2-1-1 Aoe, Kitaku, Okayama, 700-8607 Japan

**Keywords:** Unicompartmental knee arthroplasty, Meniscus, Posterior root tear, Subchondral insufficiency fracture, Osteoarthritis

## Abstract

**Introduction:**

The outcomes of unicompartmental knee arthroplasty (UKA) in the presence and absence of medial meniscus posterior root tears (MMPRTs) have not been compared. This study compared the characteristics and clinical outcomes of patients undergoing UKA with and without MMPRTs.

**Materials and methods:**

This study analyzed 68 patients. The presence or absence of MMPRTs was evaluated using preoperative magnetic resonance imaging. Patient characteristics, clinical scores before surgery and at the final evaluation, and imaging findings were compared between patients with and without MMPRTs. Multiple regression analysis was conducted on postoperative visual analog scale (VAS)-pain scores.

**Results:**

MMPRTs were present in 64.7% (44/68) of patients. Patients with MMPRTs were significantly younger (67.8 ± 8.2 vs. 75.0 ± 7.1 years, *p* < 0.001) and had a shorter duration from the development of symptoms to the time of surgery than those without (6.8 ± 8.4 vs. 36.1 ± 38.9 months, *p* < 0.001). Component placement or lower-limb alignment did not significantly differ between the groups. Preoperative clinical scores were not significantly different between the groups; however, patients with MMPRTs showed significantly better postoperative VAS-pain scores than those without (10.0 ± 9.0 vs. 28.2 ± 26.0 points, *p* = 0.026). Multiple regression analysis of postoperative VAS-pain scores revealed the significant effect of duration from the development of symptoms to the time of surgery (*p* = 0.038).

**Conclusions:**

Patients undergoing UKA with MMPRTs were younger with less radiographic osteoarthritic changes compared to those without MMPRTs, and their postoperative VAS-pain scores were significantly superior. The duration from the development of symptoms to the time of surgery significantly influenced postoperative pain in patients undergoing UKA.

## Introduction

Patients with medial meniscus posterior root tears (MMPRTs) often experience acute posteromedial knee pain during activities of daily living (ADL) [1]. Despite this characteristic, MMPRTs are frequently missed during diagnosis [2]. If left untreated, MMPRTs can lead to rapid deterioration of knee-joint function by inducing the development of knee osteonecrosis, exacerbation of bone-marrow edema, and wear of the articular cartilage. Notably, some patients undergo knee-joint arthroplasty before being diagnosed with MMPRTs [3].

In the early phase of injury, MMPRTs with minimal osteoarthritic changes are good indications for meniscal repair, and good mid- to long-term clinical outcomes and survival rates have been reported [4, 5]. However, the repair of MMPRTs does not completely prevent the osteoarthritis progression, and hence, the indications for MMPRT repair remain undetermined [6]. Varus alignment exceeding 5° or severe cartilage damage has been reported to be poor prognostic factors after pull-out repair [7]. Therefore, MMPRTs with advanced osteoarthritis or subchondral insufficiency fracture of the medial compartment may be an indication for high tibial osteotomy (HTO), unicompartmental knee arthroplasty (UKA), or total knee arthroplasty (TKA) [8, 9]. HTO for MMPRTs has been reported to achieve good clinical outcomes, with similar outcomes observed in patients with and without MMPRTs [10, 11]. Hiranaka et al. and Innocenti et al. reported good clinical outcomes using UKA for MMPRTs [12, 13]. Tagliero et al. reported better outcomes in the MMPRT than in the non-MMPRT group, mainly following TKA [14]. Therefore, as with TKA, UKA for MMPRTs may be associated with superior clinical outcomes compared to UKA for non-MMPRTs.

To date, no studies have compared the outcomes of UKA in the presence and absence of MMPRTs. This study aimed to compare patient characteristics and clinical outcomes between the MMPRT and non-MMPRT groups of patients who underwent UKA. We hypothesized that UKA would achieve superior clinical outcomes in the MMPRT compared to the non-MMPRT group.

## Materials and methods

### Patients

**Fig. 1:**
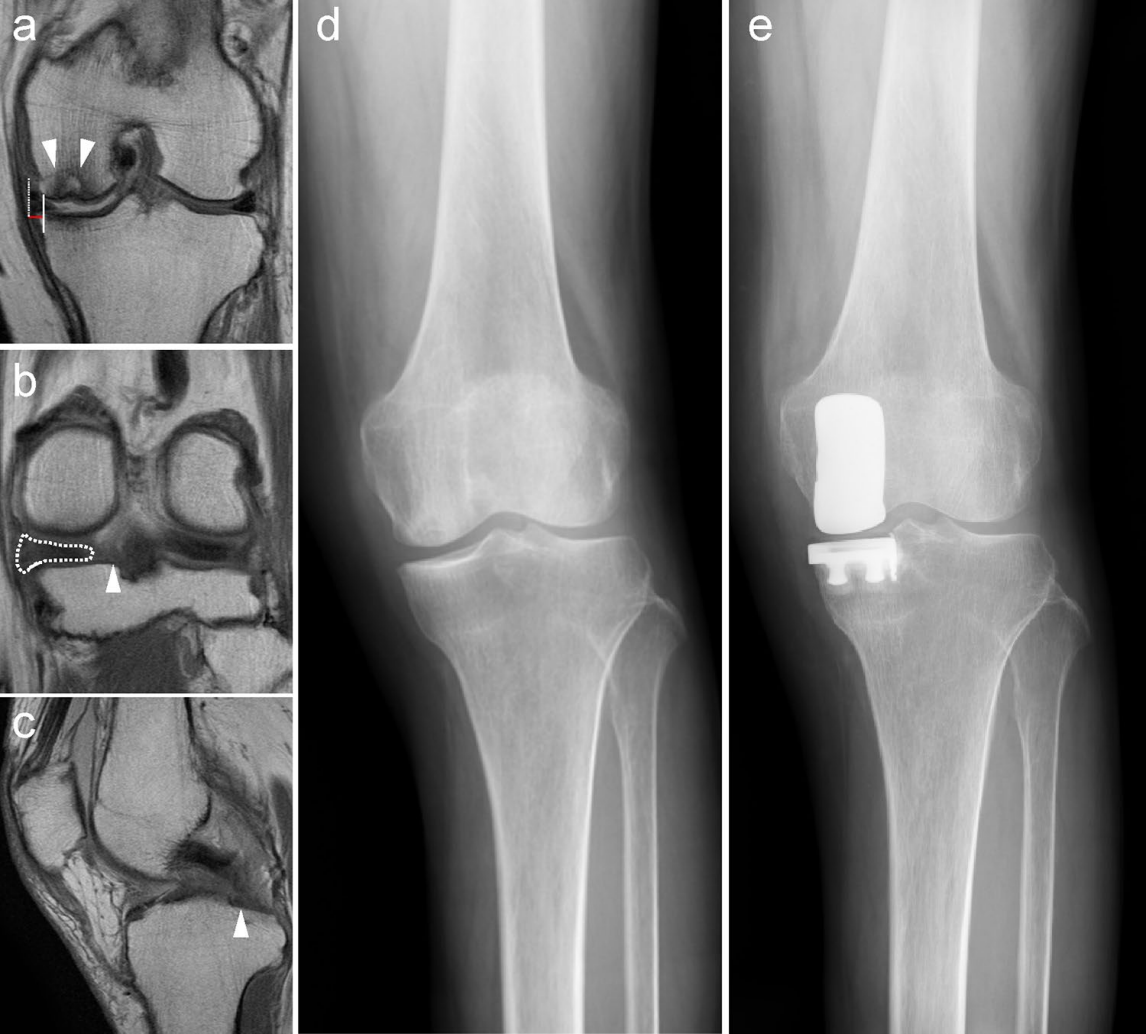
Imaging findings for patients in the MMPRT group. **a** Osteonecrosis of the distal medial femoral condyle (white arrowhead) and medial meniscus extrusion (red line) are shown (T2-weighted coronal image). **b** Cleft (white arrowhead) and giraffe neck signs (white dotted line) indicating MMPRTs are observed (T2-weighted coronal image). **c** A ghost sign (white arrowhead) indicating MMPRTs are observed (T2-weighted sagittal image). **d** Preoperative radiograph of the knee joint. **e** Postoperative radiograph of the knee joint. *MMPRT* medial meniscus posterior root tear

**Fig. 2:**
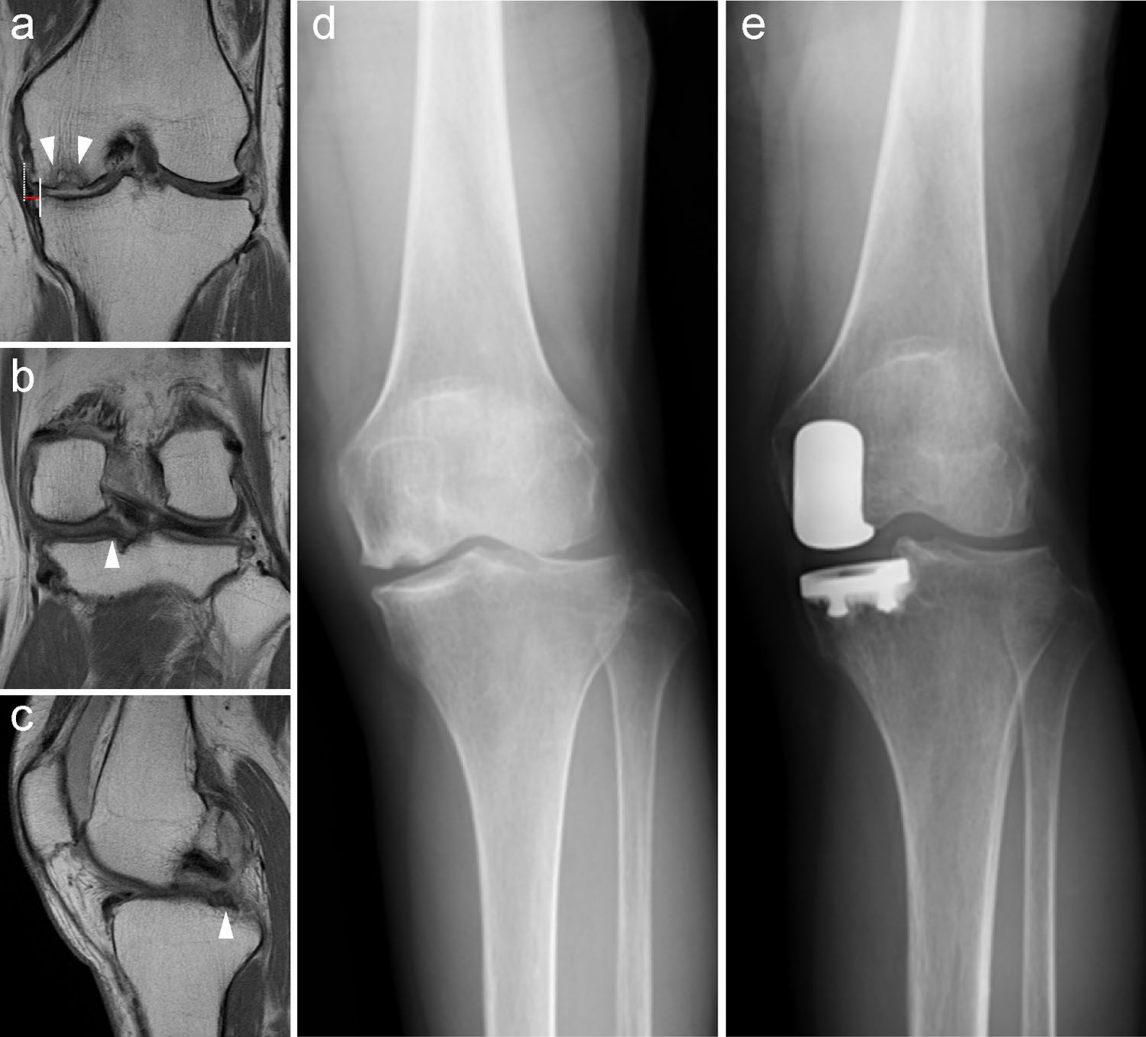
Imaging findings for patients in the non-MMPRT group. **a** Osteonecrosis of the distal medial femoral condyle (white arrow), medial meniscus extrusion (red line), and meniscus degeneration are shown (T2-weighted coronal image). **b** The continuity of the posterior root (white arrowhead) can be confirmed (T2-weighted coronal image). **c** The continuity of the posterior root (white arrowhead) can be confirmed (T2-weighted sagittal image). **d** Preoperative radiograph of the knee joint. **e** Postoperative radiograph of the knee joint. *MMPRT* medial meniscus posterior root tear

A total of 75 patients who underwent UKA between February 2018 and September 2021 were retrospectively reviewed. Six patients with a follow-up period of < 2 years and one patient without preoperative magnetic resonance imaging (MRI) scans were excluded. Finally, 68 patients were included in this study. The presence or absence of MMPRTs was evaluated using preoperative MRI scans. Patients’ characteristics, clinical scores, and imaging findings were compared between the MMPRT and non-MMPRT groups (Figs. 1, 2).

### Surgical indications

At our institution, pull-out repair is generally indicated for symptomatic acute MMPRTs. However, patients with MMPRTs with a Kellgren–Lawrence (KL) grade ≥ 3, a subchondral insufficiency fracture of the knee (SIFK) grade ≥ 3, or a femorotibial angle (FTA) > 180° are ineligible for pull-out repair. The SIFK grade was evaluated using the classification reported by Koshino et al. [15].

Our indications for UKA were: (1) knee osteoarthritis or osteonecrosis with lesions localized to the medial compartment or (2) FTA > 180° or SIFK grade ≥ 3 with irreparable MMPRTs or meniscal tears, and (3) preservation of the cartilage and meniscus in the lateral compartment on MRI. TKA was performed if these indications were absent.

### Surgical technique

The Persona Partial Knee System (Zimmer Biomet, Warsaw, IN, USA) was the implant used in all cases. Preoperatively, all patients received general anesthesia and femoral nerve block, and implantation were performed using an air tourniquet in accordance with the manufacturer’s instructions.

### Radiographic assessments

**Fig.3:**
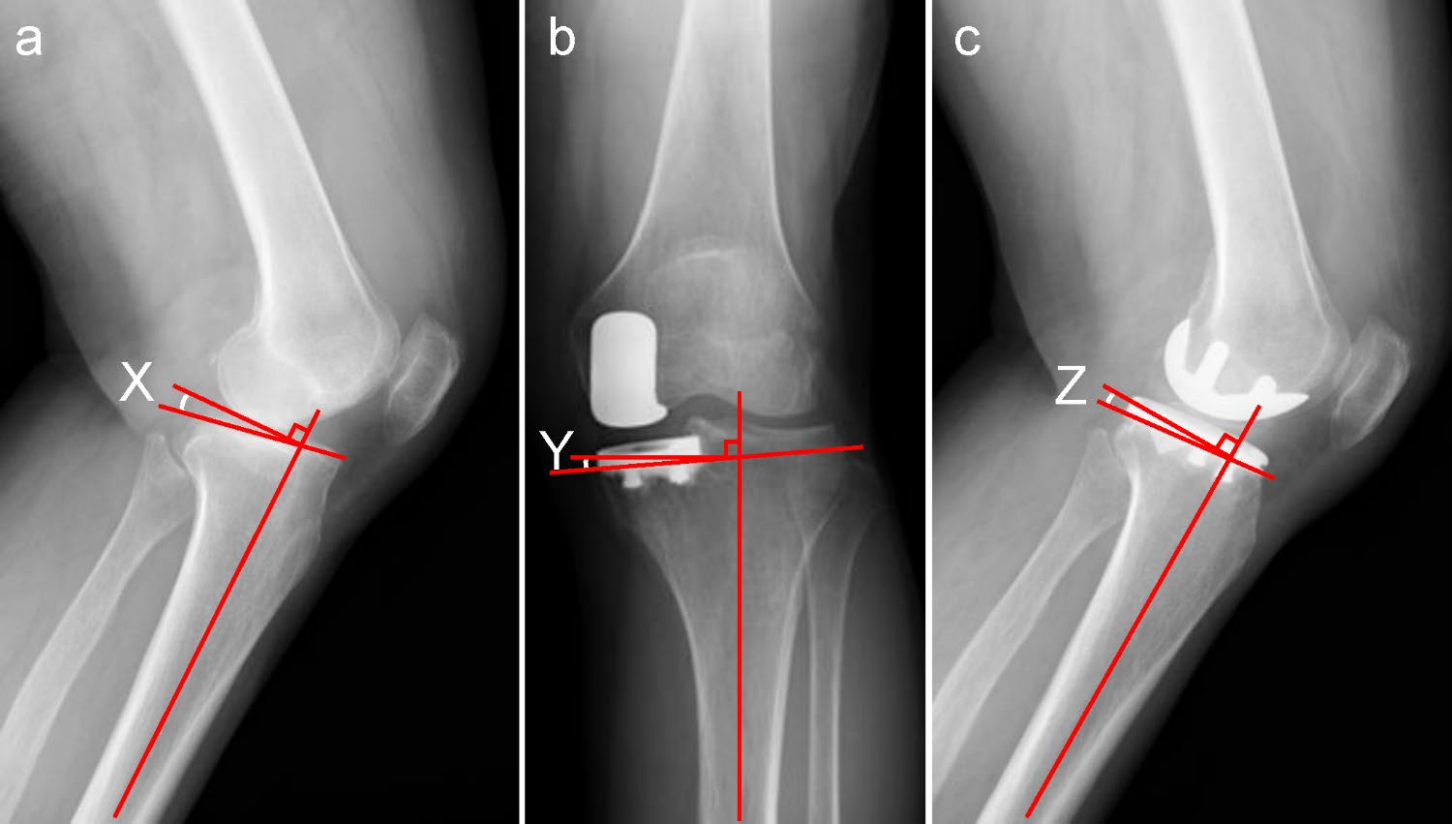
Measurement methods in radiographic assessments. **a** Preoperative PTS (angle X) was defined as the angle between the line perpendicular to the tibial bone axis and the medial tibial plateau in the lateral radiograph of the knee joint. **b** Postoperative TI (angle Y) was defined as the angle between a line perpendicular to the tibial bone axis and the inferior surface of the tibial component in the anteroposterior radiograph of the knee joint. **c** Postoperative PTS (angle Z) was defined as the angle between the line perpendicular to the tibial bone axis and the inferior surface of the tibial component in the lateral radiograph of the knee joint. *PTS* posterior tibial slope, *TI* tibial inclination

Preoperative and postoperative FTA were evaluated using a standing anteroposterior radiography of the knee joint [16]. In addition, preoperative KL grade was evaluated using the Rosenberg view of the knee joint. Preoperative and postoperative posterior tibial slope (PTS) and tibial inclination (TI) were evaluated using non-weight bearing radiography of the knee joint (Fig. 3).

### MRI assessments

Preoperative MRI scans of the knee joint were used to evaluate the presence or absence of MMPRTs, the amount of medial meniscus extrusion (MME), and SIFK grade. The presence of MMPRTs was detected based on signs such as a ghost, cleft, and giraffe neck [17]. The MME was defined as the distance from the medial margin of the tibia to that of the medial meniscus in the slice with the highest medial tibial eminence.

### Clinical scores

Clinical scores were assessed using the Knee Injury and Osteoarthritis Outcome Score (KOOS) and visual analog scale (VAS)-pain score preoperatively and at the final evaluation. The KOOS has five sub-items: pain, symptoms, ADL, quality of life, and sport and recreation function. The VAS-pain score was evaluated using a VAS that indicates the degree of pain, ranging from no pain (0) to the highest imaginable pain (100).

### Statistical analysis

Statistical analyses were performed using the EZR software (Saitama Medical Centre, Saitama, Japan). Fisher’s exact or Mann–Whitney U test was used to compare the MMPRT and non-MMPRT groups. Wilcoxon signed-rank test was used to compare the preoperative and final evaluation results for each group. Moreover, multiple regression analysis was performed on the postoperative VAS-pain scores.

To assess intra- and inter-rater reliability, FTA, PTS, and TI were measured by two independent evaluators. In the post-hoc analysis, the effect size was calculated from the mean and standard deviation of the postoperative VAS-pain scores in the two groups, and the statistical power was evaluated with statistical significance set at *p* = 0.05 (G*Power, University of Dusseldorf, Dusseldorf, Germany).

## Results

Table 1 summarizes the patients’ demographic and clinical characteristics. MMPRTs were present in 44 (64.7%) of 68 patients. The age at surgery was significantly lower in the MMPRT than in the non-MMPRT group (67.8 ± 8.2 vs. 75.0 ± 7.1 years, *p* < 0.001). The duration from the development of symptoms to the time of surgery was significantly shorter in the MMPRT than in the non-MMPRT group (6.8 ± 8.4 vs. 36.1 ± 38.9 months, *p* < 0.001).

**Table 1:** Patient characteristics

	MMPRT group	Non-MMPRT group	*p*-value
Patients, *n* (%)	44 (64.7%)	24 (35.3%)	
Sex (male/female), *n*	5/39	4/20	0.710
Age, years	67.8 ± 8.2	75.0 ± 7.1	< 0.001*
Height, m	1.54 ± 0.08	1.53 ± 0.09	0.460
Body weight, kg	59.5 ± 11.5	58.8 ± 8.6	0.944
Body mass index, kg/m^2^	25.0 ± 3.6	25.2 ± 2.7	0.608
Duration from the development of symptoms to the time of surgery, months	6.8 ± 8.4	36.1 ± 38.9	< 0.001*
Follow-up duration, months	32.5 ± 8.7	35.5 ± 11.7	0.396

Data are presented as mean ± standard deviation or number. Statistical analyses were performed using Fisher’s exact test or Mann–Whitney U test

*MMPRT* medial meniscus posterior root tear

*Statistically significant

Table 2 presents a comparison of radiographic and MRI evaluation results. No significant differences were observed in the preoperative FTA, PTS, KL grade, and SIFK grade and postoperative FTA, PTS, and TI between the two groups.

**Table 2:** Comparison of radiographic and MRI evaluation results

	MMPRT group	Non-MMPRT group	*p*-value
Preoperative FTA, °	179.5 ± 2.1	180.4 ± 2.2	0.236
Preoperative PTS, °	10.6 ± 2.4	10.4 ± 2.1	0.755
Preoperative KL grade (0/1/2/3/4), *n*	0/1/16/25/2	0/0/8/11/5	0.187
Preoperative KL grade (≥ 3), *n* (%)	27 (61.4%)	16 (66.7%)	0.794
Preoperative SIFK grade (0/1/2/3/4), *n*	16/11/2/10/3	11/3/5/2/3	0.104
Preoperative SIFK grade (≥ 3), *n* (%)	13 (29.5%)	5 (20.8%)	0.569
Preoperative MME, mm	5.82 ± 1.59	3.79 ± 1.59	< 0.001*
Postoperative FTA, °	177.1 ± 2.0	177.2 ± 1.9	1.000
Postoperative PTS, °	6.0 ± 1.8	6.4 ± 2.5	0.731
Postoperative TI, °	2.4 ± 2.2	2.5 ± 1.5	0.532

Data are presented as mean ± standard deviation or number. Statistical analyses were performed using Fisher’s exact test or Mann–Whitney U test

FTA, femorotibial angle; KL, Kellgren–Lawrence; MME, medial meniscus extrusion; MMPRT, medial meniscus posterior root tear; MRI, magnetic resonance imaging; PTS, posterior tibial slope; SIFK, subchondral insufficiency fracture of the knee; TI, tibial inclination

*Statistically significant

Clinical scores significantly improved in both groups at the final evaluation (*p* < 0.001; Table 3). The preoperative clinical scores did not significantly differ between the two groups (Table 4). The postoperative VAS-pain scores were significantly lower in the MMPRT than in the non-MMPRT group (10.0 ± 9.0 vs. 28.2 ± 26.0 points, *p* = 0.026).

**Table 3:** Comparison of preoperative and postoperative clinical scores in the MMPRT and non-MMPRT groups

	MMPRT group			Non-MMPRT group		
	Preoperative	Postoperative	*p*-value	Preoperative	Postoperative	*p*-value
KOOS-pain	52.1 ± 18.8	87.5 ± 9.9	< 0.001*	47.1 ± 18.4	85.1 ± 13.5	< 0.001*
KOOS-symptoms	53.9 ± 20.3	86.5 ± 10.2	< 0.001*	52.8 ± 16.0	84.3 ± 15.6	< 0.001*
KOOS-ADL	63.2 ± 16.0	88.9 ± 10.5	< 0.001*	56.4 ± 19.4	84.7 ± 14.8	< 0.001*
KOOS-sport/rec	23.5 ± 23.1	50.6 ± 30.9	< 0.001*	19.8 ± 16.8	46.7 ± 37.0	< 0.001*
KOOS-QOL	32.7 ± 19.9	68.4 ± 17.3	< 0.001*	27.5 ± 13.0	65.8 ± 22.4	< 0.001*
VAS pain score	48.0 ± 24.7	10.0 ± 9.0	< 0.001*	51.0 ± 17.8	28.2 ± 26.0	< 0.001*

Data are presented as mean ± standard deviation. Statistical analyses were performed using Wilcoxon signed–rank test

ADL, activities of daily living; KOOS, Knee Injury and Osteoarthritis Outcome Score; MMPRT, medial meniscus posterior root tear; QOL, quality of life; sport/rec, sport and recreation function; VAS, visual analog scale

*Statistically significant

**Table 4:** Comparison of preoperative and postoperative clinical scores between the MMPRT and non-MMPRT groups

	MMPRT group	Non-MMPRT group	*p*-value
Preoperative			
KOOS-pain	52.1 ± 18.8	47.1 ± 18.4	0.480
KOOS-symptoms	53.9 ± 20.3	52.8 ± 16.0	0.878
KOOS-ADL	63.2 ± 16.0	56.4 ± 19.4	0.230
KOOS-sport/rec	23.5 ± 23.1	19.8 ± 16.8	0.918
KOOS-QOL	32.7 ± 19.9	27.5 ± 13.0	0.406
VAS pain score	48.0 ± 24.7	51.0 ± 17.8	0.326
Postoperative			
KOOS-pain	87.5 ± 9.9	85.1 ± 13.5	0.689
KOOS-symptoms	86.5 ± 10.2	84.3 ± 15.6	0.959
KOOS-ADL	88.9 ± 10.5	84.7 ± 14.8	0.378
KOOS-sport/rec	50.6 ± 30.9	46.7 ± 37.0	0.728
KOOS-QOL	68.4 ± 17.3	65.8 ± 22.4	0.462
VAS pain score	10.0 ± 9.0	28.2 ± 26.0	0.026*

Data are presented as mean ± standard deviation. Statistical analyses were performed using Mann–Whitney U test

ADL, activities of daily living; KOOS, Knee Injury and Osteoarthritis Outcome Score; MMPRT, medial meniscus posterior root tear; QOL, quality of life; sport/rec, sport and recreation function; VAS, visual analog scale

*Statistically significant

Multiple regression analysis of postoperative VAS-pain scores revealed the significant effect of duration from the development of symptoms to the time of surgery (*p* = 0.038; Table 5). The intra-/inter-rater reliability of FTA, PTS, and TI were 0.973/0.978, 0.919/0.895, and 0.931/0.899, respectively. In the post-hoc analysis, the statistical power was 93.5% at *p* = 0.05.

**Table 5:** Multiple regression analysis of postoperative VAS pain scores

Variables	*Β*	*t*-value	*p*-value	95% confidence interval
(Intercept)	− 53.059	− 0.241	0.811	− 493.836 to 387.719
Age	0.469	1.772	0.081	− 0.060 to 0.998
Duration from the development of symptoms to the time of surgery	0.176	2.116	0.038*	0.010–0.342
Preoperative FTA	1.497	1.177	0.244	− 1.045 to 4.038
Postoperative FTA	− 1.330	− 0.974	0.334	− 4.061 to 1.400

Statistical analyses were performed using multiple regression analysis

FTA, femorotibial angle; VAS, visual analog scale; β, partial regression coefficient

*Statistically significant

## Discussion

The most important findings of the present study were that 64.7% of patients who underwent UKA had MMPRTs, and that compared to the non-MMPRT group, the MMPRT group had superior postoperative VAS-pain scores after UKA.

Recently, spontaneous osteonecrosis of the knee has been considered to result from mechanical overload of the subchondral bone due to meniscal injury or dysfunction, and has been termed SIFK [18]. The SIFK has been reported to have a strong relationship with MMPRTs, and 62–80% of patients with SIFK have MMPRTs [19, 20]. Wu et al. reported that MMPRTs were present in 69.6% of the patients who underwent UKA [21]. In the present study, 64.7% of patients who underwent UKA and 72.2% of those with SIFK grade ≥ 3 had MMPRTs; these results were comparable to those reported previously.

Several factors have been reported to affect the postoperative clinical outcomes of UKA, including component placement, lower-limb alignment, and PTS [22, 23]. Age, body mass index, osteoarthritis grade, and bone-marrow edema are not associated with the postoperative outcomes of UKA [24–27]. In this study, no significant differences in the component placement, lower-limb alignment, and PTS were observed between the MMPRT and non-MMPRT groups. Therefore, the difference in postoperative VAS-pain scores between the two groups may have been largely unaffected by component placement or lower-limb alignment.

MMPRTs are characterized by sudden onset of pain and short-term progression of osteoarthritis due to disruption of the hoop function of the meniscus [28]. In contrast, the progression of osteoarthritis in patients without MMPRTs is relatively slow, although the causes vary [29]. Previous studies have reported the effects of pain duration and central sensitization on the risk of residual postoperative pain after knee surgery [30]. In the present study, the preoperative VAS-pain scores in the MMPRT and non-MMPRT groups were similar. In contrast, the postoperative VAS-pain scores were worse in the non-MMPRT group than in the MMPRT group. The MMPRT and non-MMPRT groups showed significant differences in patient characteristics in terms of the duration from the development of symptoms to the time of surgery. Furthermore, multiple regression analysis of the postoperative VAS-pain scores revealed the significant effect of duration from the development of symptoms to the time of surgery. These findings suggest that the reason for the difference in postoperative VAS-pain scores between the two groups may be chronic pain and associated central sensitization due to the longer duration from the development of symptoms to the time of surgery. In patients undergoing UKA, especially patients with chronic pain such as those without MMPRTs, the effect of central sensitization should be considered, and attention should be paid to surgical indications and pain control during the preoperative period.

In this study, the VAS-pain score showed a significant difference between patients with and without MMPRTs. Conversely, no significant difference in the KOOS-pain score was found. The KOOS-pain score evaluates the degree of pain during each activity, whereas the VAS-pain score assesses the most intense and unbearable pain ever experienced as the maximum value and may therefore reflect more psychological influences.

Among 68 patients who underwent UKA in this study, 25 patients had a KL grade of ≤ 2. These patients underwent UKA because they had FTA > 180° or SIFK grade ≥ 3. Some studies have reported that UKA is indicated in patients with bone-on-bone arthritis [31]. In our institution, patients with poor alignment or severe cartilage damage, which are poor prognostic factors for the repair of MMPRTs or other meniscal tears, are not considered as candidates for meniscal repair. This may be the reason why many patients with early-stage osteoarthritis with low KL grade were included in this study. Innocenti et al. reported favorable outcomes in patients undergoing UKA with MMPRTs and mild radiographic osteoarthritis changes [13]. They also reported that patients with MMPRTs, even those with mild osteoarthritis changes without bone-on-bone findings such as KL grades 1–3, have preoperative pain equivalent to or greater than that of patients with end-stage osteoarthritis with bone-on-bone findings such as KL grade 4 [13]. The key point of surgical indication for UKA is to confirm that the knee joint pain is associated with a lesion in the unicompartmental knee joint and not the occurrence of bone-on-bone findings.

This study has some limitations. First, it was a retrospective study. Second, the average follow-up period was approximately three years, which was short. Third, we did not perform long-leg radiography in this study; this could have impacted the lower-limb alignment assessment. Fourth, the patients were not stratified; therefore, the effect of age difference between the two groups on the postoperative scores cannot be completely dismissed. Finally, the effects of chronic pain and central sensitization were not evaluated using scores. Future evaluations to assess the impact of chronic pain and central sensitization may help to determine the appropriate indications for UKA.

## Conclusion

In this study, MMPRTs were present in 64.7% of patients who underwent UKA. Compared to the non-MMPRT group, the MMPRT group included younger, who had less radiographic osteoarthritic changes, and superior postoperative VAS-pain scores after UKA. The duration from the development of symptoms to the time of surgery significantly influenced postoperative pain in patients who underwent UKA.

## Data Availability

The data that support the findings of this study are available from the corresponding author, upon reasonable request. Medial meniscus posterior root tears with advanced osteoarthritis or subchondral insufficiency fracture are good indications for unicompartmental knee arthroplasty at a minimum 2-year follow-up.
